# Diagnostic Accuracy of Calretinin for Malignant Mesothelioma in Serous Effusions: a Meta-analysis

**DOI:** 10.1038/srep09507

**Published:** 2015-03-30

**Authors:** Diandian Li, Bo Wang, Hongyu Long, Fuqiang Wen

**Affiliations:** 1 Department of Respiratory Medicine,West China Hospital of Sichuan University, Guoxuexiang 37, Chengdu, Sichuan, China; 2 Division of Pulmonary Diseases, State Key Laboratory of Biotherapy of China, Sichuan University, No.1 4th Keyuan Road, Chengdu, Sichuan, China

## Abstract

Numerous studies have investigated the utility of calretinin in differentiating malignant mesothelioma (MM) from metastatic carcinoma (MC) in serous effusions. However, the results remain controversial. The aim of this study is to determine the overall accuracy of calretinin in serous effusions for MM through a meta-analysis of published studies. Publications addressing the accuracy of calretinin in the diagnosis of MM were selected from the Medline (Ovid), PubMed, the Cochrane Library Database and the Web of Science. Data from selected studies were pooled to yield summary sensitivity, specificity, positive and negative likelihood ratio (LR), diagnostic odds ratio (DOR), and receiver operating characteristic (SROC) curve. Statistical analysis was performed by Meta-Disc 1.4 and STATA 12.0 softwares. 18 studies met the inclusion criteria and the summary estimating for calretinin in the diagnosis of MM were: sensitivity 0.91 (95%*CI*: 0.87–0.94), specificity 0.96 (95%*CI*: 0.95–0.96), positive likelihood ratio (PLR) 14.42 (95%*CI*: 7.92–26.26), negative likelihood ratio (NLR) 0.1 (95%*CI*: 0.05–0.2) and diagnostic odds ratio 163.03 (95%*CI*: 54.62–486.63). The SROC curve indicated that the maximum joint sensitivity and specificity (Q-value) was 0.92; the area under the curve was 0.97. Our findings suggest that calretinin may be a useful diagnostic tool for confirming MM in serous effusions.

Effusion in pleural and peritoneal cavities is a common complication of malignancies such as lung, breast, gastrointestinal and female genital adenocarcinoma as well as malignant mesothelioma^1^,^2^. Reliable identification of the primary tumor origin is very important for staging and has significant treatment implications. However, distinction between malignant mesothelioma (MM) and adenocarcinoma involving the serous membranes is one of the most challenging diagnostic problems in surgical pathology and effusion cytology^3^. There are no reliable cytomorphological features to differentiate MM cells from metastatic carcinoma (MC). Although a biopsy provides a relatively high sensitivity and has been used as the gold standard diagnostic method^4^,^5^, these operations are invasive, operator dependent, and may complicate subsequent disease management by seeding tumor cells or be unfeasible because of poor condition of the patient. Tumor biomarkers provide significant help in this differential diagnosis and are increasingly attractive because of their noninvasive feature and relative inexpensiveness. Hence, many antibodies directed against specific cell type antigens have been used in serous effusions as tumor markers to improve the diagnostic accuracy^6^,^7^, but it remains unclear which marker has a superior performance or which antibodies compose the optimum panel of diagnostic markers for early and accurate detection of MM.

Calretinin, a 29-kD calcium-binding protein, is expressed normally in neurons of the central and peripheral nervous system^8^. An increasing number of studies have shown the ability of this antibody as a biomarker for the diagnosis of MM in effusion specimens^9^,^10^,^11^,^12^,^13^,^14^,^15^,^16^,^17^,^18^,^19^,^20^,^21^,^22^,^23^,^24^,^25^,^26^. Systematic analysis of these data may be valuable to finally confirm the application potential of calretinin as a marker for MM. Therefore, in current study, we performed a meta-analysis to summarize the literature on the overall accuracy of calretinin for differentiating MM from MC in serous effusions.

## Methods

### Search strategy and study selection

We searched the following electronic databases: Medline (Ovid), PubMed, the Chinese Journals Full-text Database (CNKI), the Cochrane Library Database and the Web of Science (updated to December 31, 2013) to identify articles evaluating the diagnostic value of calretinin for MM in serous effusions. The search terms used were: “calretinin,” “body fluids,” “effusions,” “sensitivity and specificity,” and “accuracy.” Only full-text papers published in English and Chinese were included. The reference lists of identified articles were checked to obtain additional relevant articles. Studies were included if they met all of the following criteria: (1) studies evaluated calretinin in the differential diagnosis of MM and MC in serous effusions, (2) each study contains more than ten fluid specimens, and (3) studies must provide sufficient data to calculate both sensitivity and specificity. Conference abstracts and letters to the journal editors were excluded because of the limited data presented in them. Two reviewers (DDL and BW) independently judged study eligibility while screening the citations. Disagreements were resolved by consensus.

### Data extraction and quality assessment

Two independent authors (BW and DDL) extracted the data and reached a consensus on all items. Any disagreements were resolved by discussion with a third author to reach a final consensus. Data retrieved from the reports included the first author's name, publication year, country, test methods, cutoff value, sensitivity, specificity, and methodological quality. The methodological quality of each study was assessed by QUADAS (quality assessment for studies of diagnostic accuracy, an evidence-based quality assessment tool for use in systematic reviews of diagnostic accuracy studies, maximum score 14)^27^.

### Statistical analyses

The standard methods recommended for the diagnostic accuracy of meta-analyses were used^28^. The following measures of test accuracy were computed for each study: sensitivity, specificity, positive likelihood ratio (PLR), negative likelihood ratio (NLR), and diagnostic odds ratio (DOR). The diagnostic threshold identified for each study was used to plot a summary receiver operating characteristic (SROC) curve^29^. To detect cut-off threshold effects, the relationship between sensitivity and specificity was evaluated by the Spearman correlation coefficient. The inter-study heterogeneity was calculated by the chi-square-based Q test and the inconsistency index *I^2^*. When a significant Q test (*p* < 0.05 or *I^2^* > 50%) indicated heterogeneity among studies, the random-effect model (DerSimonian–Laird method) was conducted for the meta-analysis to calculate the pooled sensitivity, specificity, and other related indexes of the studies; otherwise, the fixed-effect model (Mantel–Haenszel method) was chosen. Meta-regression was performed to investigate the source of heterogeneity within the included studies (inverse variance weighted)^30^. Subgroup analyses were also performed if necessary to dissect the heterogeneity. Since publication bias is of concern for meta-analyses of diagnostic studies, we tested for the potential presence of this bias using Deeks' funnel plots^31^. Analyses were performed using the following statistical software programs: STATA, version 12.0 (Stata Corporation, College Station, TX, USA) and Meta-Disc 1.4 for Windows (XI Cochrane Colloquium, Barcelona, Spain)^32^,^33^. In every test, a two-sided *p*-value of <0.05 was considered statistically significant.

## Results

### Characteristics and quality of the included studies

**Figure 1:**
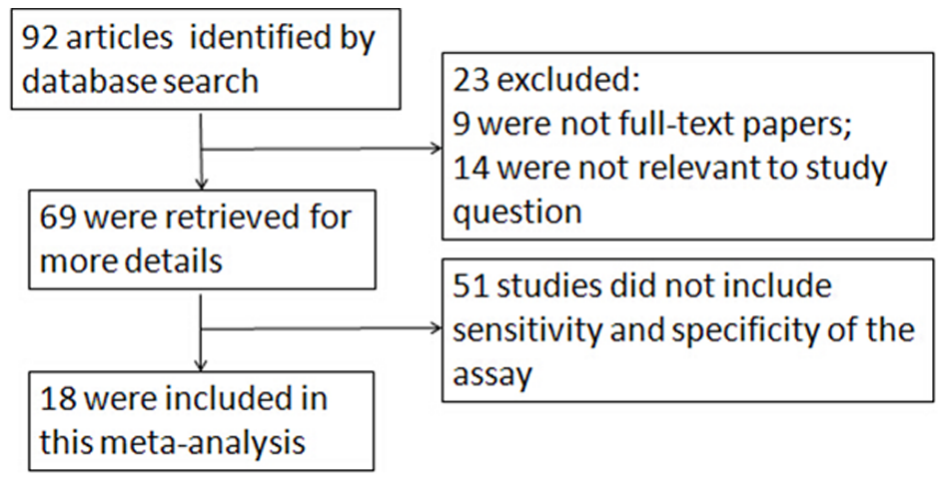
Flow chart of selection process for eligible articles.

The article selection process used in this study is summarized in Fig. 1. The meta-analysis was performed on the final 18 studies. The main clinical characteristics of the included studies are presented in Table 1. Overall, the 18 selected studies, which originated from 7 countries, included 2276 individuals and the sample size varied from 30 to 1158 individuals with an average size of 126 individuals. In all studies included in the meta-analysis, the cytological diagnoses of all cases were proved by histopathology or clinical data.

### Diagnostic accuracy

The threshold effect is caused by differences of sensitivity and specificity. In this meta-analysis, the Spearman correlation coefficient of sensitivity and 1-specificity was −0.476 with a *p*- value of 0.06, suggesting that there is no heterogeneity from threshold effect. The between-study heterogeneity was assessed by *I^2^* index to choose the appropriate calculation model. The *I^2^* of sensitivity, specificity, positive likelihood ratio (PLR), negative likelihood ratio (NLR) and DOR were 59.1% (*p* = 0.0008), 81.1% (*p* <0.0001), 84.9% (*p* <0.0001), 67.9% (*p* <0.0001), and 71.7% (*p* <0.0001), respectively. Therefore, the random effects model was used for calculating pooled sensitivity, specificity, PLR, NLR and DOR.

**Figure 2:**
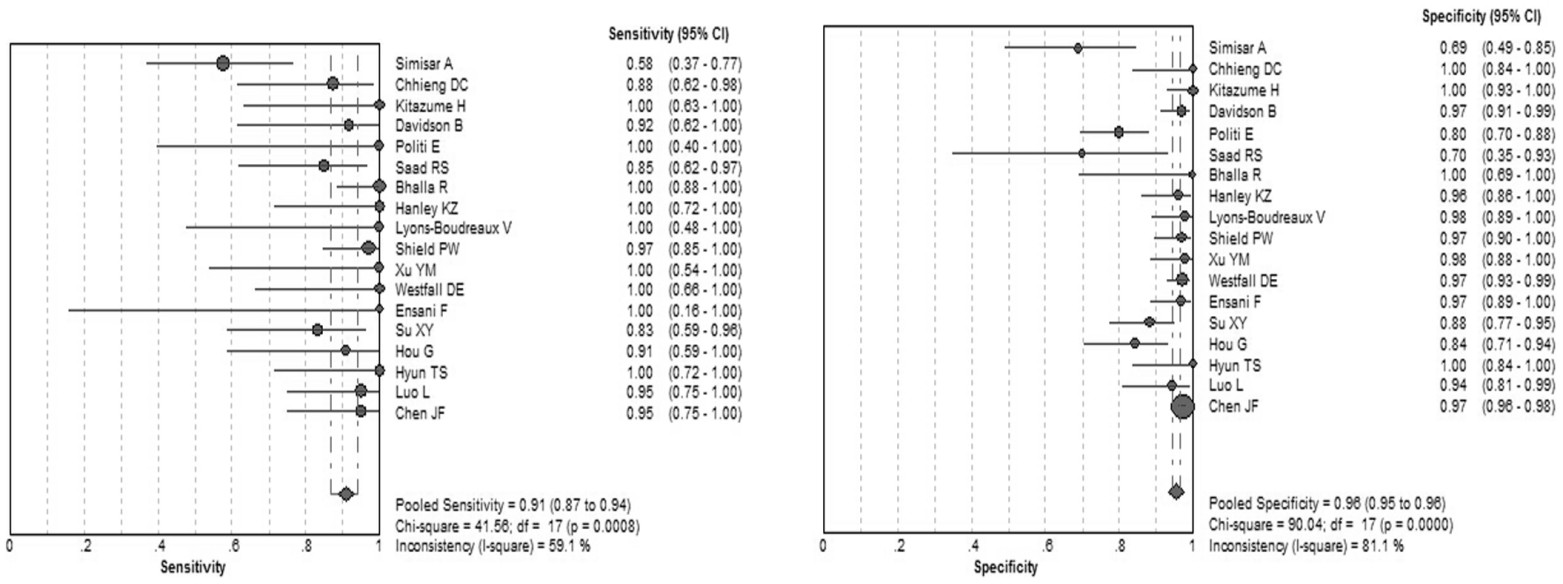
Forest plots of the sensitivity and specificity for calretinin in the diagnosis of malignant mesothelioma for all studies. The point estimates of sensitivity and specificity for each study are shown as solid circles and the size of each solid circle indicates the sample size of each study. Error bars are 95% confidence intervals.

**Figure 3:**
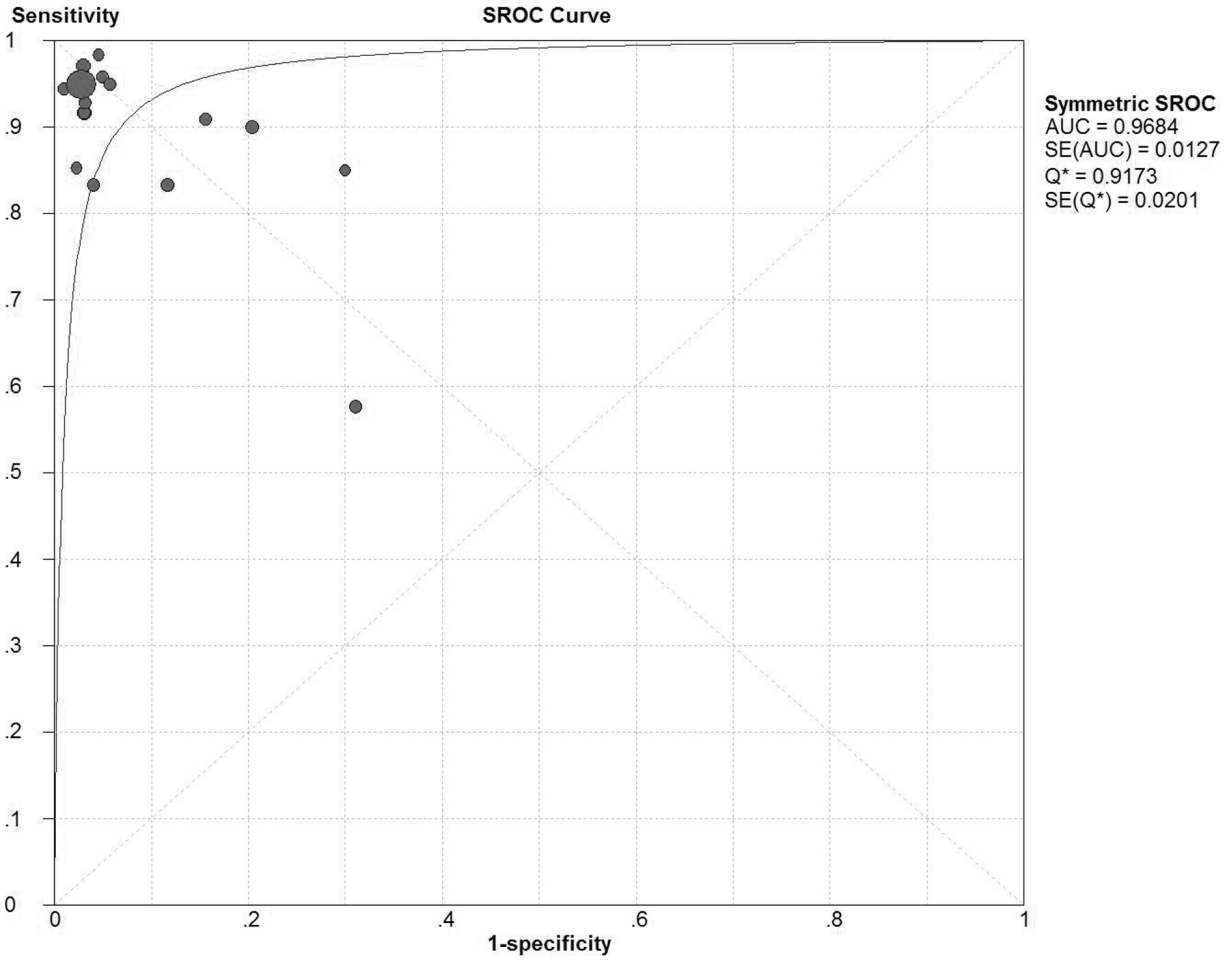
Summary receiver operating characteristic (SROC) curve for calretinin in the diagnosis of malignant mesothelioma for all studies. Solid circles represent each study included in the meta-analysis. The size of each solid circle indicates the size of each study. The regression SROC curve summarizes the overall diagnostic accuracy.

Fig. 2 displays the forest plots of the sensitivity and specificity of these 18 studies concerning calretinin in the diagnosis of MM. The pooled sensitivity and specificity were 0.91 (95%*CI*: 0.87–0.94) and 0.96 (95%*CI*: 0.95–0.96), respectively. The overall PLR and NLR were 14.42 (95%*CI*: 7.92–26.26) and 0.1 (95%*CI*: 0.05–0.2), respectively. The pooled diagnostic odds ratio (DOR) was 163.03 (95%*CI*: 54.62–486.63). The SROC curve for calretinin is shown in Fig. 3, which indicates sensitivity versus 1-specificity of individual studies. As a global measure of test efficacy we used Q-value, the intersection point of the SROC curve with a diagonal line from the left upper corner to the right lower corner of the ROC space which corresponds to the highest common value of sensitivity and specificity for the test, for the overall measure of the discriminatory power of the test. Our data showed that the SROC curve for calretinin is positioned near the desirable upper left corner and the Q-value was 0.92; while the area under the curve (AUC) was 0.97, indicating that the level of overall accuracy was high.

### Meta-regression analysis

To explore the possible reasons for the heterogeneity, a meta-regression analysis based on method (cell blocks or smears), sample size (≥100 or <100), geographical location (America, Europe or Asia) and QUADAS scores (≥10 or <10) were performed. In the present study, none of the above covariates included in the meta-regression were found to be the significant source of heterogeneity (all *p* > 0.05) (Table 2).

### Sensitivity analyses

As consistent staining pattern of antibody is important for diagnostic test, sensitivity analyses were performed based on different cut-off staining patterns. 14 studies^10^,^11^,^13^,^14^,^15^,^16^,^17^,^18^,^19^,^20^,^21^,^22^,^23^,^25^ reported test results with the cut-off of presenting nuclear staining. The pooled sensitivity and specificity were 0.94 (95% CI: 0.89–0.97) and 0.94 (95% CI: 0.91–0.95), respectively, indicating a slightly higher diagnostic accuracy than the overall analysis (Table 3). We also performed a sensitivity analysis for studies^9^,^14^,^19^,^20^,^23^,^24^,^26^ that reported diagnostic accuracy of calretinin for MM in pleural effusions. The results showed that the summary sensitivity and specificity were 0.84 (95% CI: 0.76–0.91) and 0.96 (95% CI: 0.95–0.97), respectively, indicating a slightly lower diagnostic accuracy compared with the overall analysis in all included patients (Table 3).

### Publication bias evaluation

**Figure 4:**
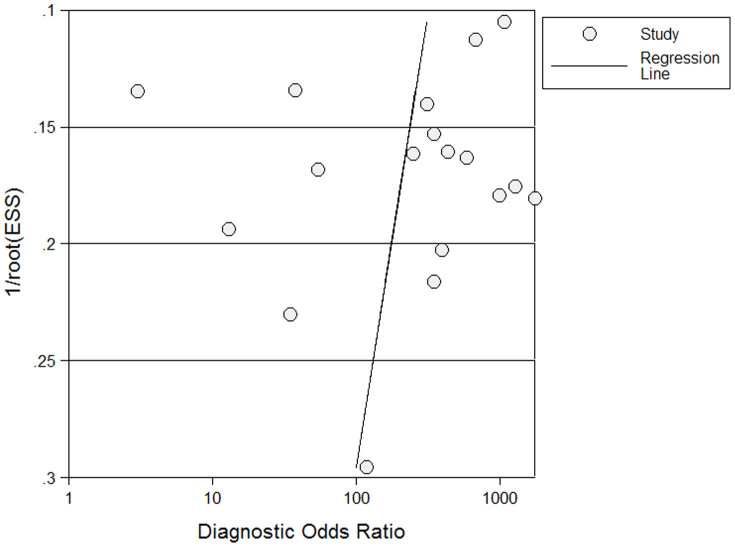
Funnel graph for the assessment of potential publication bias of the 18 included studies. The funnel graph plots the log of the diagnostic odds ratio (DOR) against the standard error of the log of the DOR (an indicator of sample size). Solid circles represent each study in the meta-analysis. The line indicates the regression line.

Publication bias was explored through Deeks' funnel plots. The shape of the funnel plot of the pooled DOR of calretinin for the diagnosis of MM did not reveal any evidence of obvious asymmetry (Fig. 4). The Deeks' test also showed a statistically non-significant value (*p* = 0.208), indicating that there was no potential publication bias.

## Discussion

Reliable conformation of a diagnosis of malignancy in effusion specimens in patients with unknown primary cancer is crucial for treatment and prognosis. However, distinguishing MM from MC in serous effusions is often difficult, and sometimes impossible, when based on morphological criteria alone. Immunocytochemistry (ICC) can provide helpful information to supplement the cytomorphology. Nevertheless, currently available markers have varying sensitivities and specificities for epithelial or mesothelial cells. Calretinin is a 29 kDa calcium-binding protein expressed in central and peripheral neural tissue, as well as in a range of other normal tissue including mesothelium, endometrial and adrenal cortical cells. As calretinin is strongly reactive in benign and malignant mesothelial cells imparting a cytoplasmic and nuclear staining pattern, it has proved to be a useful ICC marker for distinguishing malignant or reactive mesothelial cells from adenocarcinoma cells^34^. In recent years, an increasing number of studies have attempted to assess the diagnostic utility of calretinin for MM but the results remain controversial because of several factors, including the differences in study designs, sample size, statistical methods, etc.^35^. Thus, we performed the current meta-analysis to comprehensively evaluate the differential diagnostic accuracy of calretinin for MM in serous effusions.

The SROC curve presents a global summary of test performance, and shows the trade-off between sensitivity and specificity. An AUC value of more than 0.97 indicates excellent accuracy^36^. The present meta-analysis has shown that the pooled sensitivity of the calretinin was 0.91 while the pooled specificity was 0.96, and that the maximum joint sensitivity and specificity (Q value) was 0.92 while the AUC was 0.97, indicating a very good overall accuracy in the diagnosis of MM, although not perfect. The DOR, the ratio of the odds of positivity in disease relative to the odds of positivity in the non-diseased, is a single indicator of diagnostic test performance^37^ that combines the strengths of sensitivity and specificity as prevalence in dependent indicators. The value of a DOR ranges from 0 to infinity, with higher values indicating better discriminatory test performance (higher accuracy). A DOR of 1.0 indicates that a test cannot discriminate between patients with the disorder and those without it. In this meta-analysis, the pooled DOR was 163.03, also suggesting a high level of overall accuracy. However, the SROC curve and the DOR are not easy to interpret and use in clinical practice, while the likelihood ratio (PLR and NLR) is more clinically meaningful for our measures of diagnostic accuracy. A PLR value of 14.42 suggests that patients with MM have about 14-fold higher chance of being calretinin-positive compared to those with MC, and this was high enough for the clinical practice. On the other hand, the NLR was 0.1, which means that the probability of having MM in calretinin-negative patients is 10% in theory, while, for instance, malignant mesothelial cells may be absent or scanty on the cell blocks or smears used for immunostaining, which may have inflated the false negative rate.

Heterogeneity is a potential problem when interpreting the results for all meta-analysis. The *I^2^* test for the pooled specificity and PLR indicated that the heterogeneity between the studies was obvious. As threshold effect is one of the major causes of heterogeneity in test accuracy studies due to different cut-offs, we used the Spearman correlation coefficient to analyze the threshold effect. The result showed no correlation between sensitivity and specificity (p > 0.05), suggesting that threshold effect is not the source of the heterogeneity. So we undertook a meta-regression analysis to find other possible reasons for heterogeneity, including method (cell blocks or smears), sample size (≥100 or <100), geographical location (America, Europe or Asia) and QUADAS scores (≥10 or <10). In our meta-analysis, none of the variables included in the meta-regression analysis were observed to substantially affect the diagnostic accuracy of calretinin for MM.

In order to improve the homogeneity of the results, we performed sensitivity analyses based on staining pattern and effusion type. When a positive result for calretinin is recorded when nuclear staining is presented, the sensitivity analysis results were similar to the overall results with slightly increased sensitivity and decrease specificity, further proving the utility of calretinin in MM differential diagnosis. In addition, different sample origins may influence the diagnostic accuracy of calretinin for MM. Thus, we also performed a sensitivity analysis by focusing on studies that reported diagnostic accuracy parameters in pleural effusions. The summary results showed lower diagnostic utility of calretinin for MM in pleural effusions, compared with the overall results. However, due to the limited data provided in original articles, diagnostic value of calretinin for peritoneal or pericardial MM could not be synthesized by meta-analysis. Therefore, further studies are needed to evaluate and compare the diagnostic accuracy of calretinin for different type of serous effusions.

In this meta-analysis, the results indicate that calretinin may, to a certain extent, be valuable in the differential diagnosis between MM and MC in serous effusions. However, no single antibody could establish the diagnosis in all cases of body cavity fluid, and combinations of calretinin with other mesothelial or epithelial stains are recommended to increase diagnostic accuracy^13^. In recent years, some newer ICC stains including D2-40, WT-1, podoplanin, and X-linked inhibitor of apoptosis (XIAP) have been investigated as potentially effective markers for mesothelial cells^34^. Many studies showed that these markers were useful in detecting mesothelial cell lesions and distinguishing them from epithelial cells^34^,^38^,^39^,^40^,^41^. In addition, Lozano et al. has reported that some epithelial markers such as MOC-31 and Ber-EP4 were very useful with a high ability to distinguish epithelial and mesothelial cells^42^. The staining combination of positive for MOC-31 and negative for D2-40 or calretinin was 100% specific and 99% sensitive for MM^43^. However, due to the varying degrees of diagnostic accuracy of identical markers reported in different studies, it remains unclear which marker has a superior performance. Therefore, more immunomarkers should be comprehensively evaluated for their diagnostic accuracy and high-quality diagnostic tests are needed to find the optimum panel of antibodies for the diagnosis of MM in serous effusions. Moreover, few publications have evaluated diagnostic value of calretinin for differentiating different types of MM from MC in serous effusions. It has been reported by Rakha EA, et al that cytologic diagnostic accuracy in pleural effusions of epithelioid, sarcomatoid and biphasic MM were significantly different, with epithelioid MM providing the highest sensitivity while sarcomatoid type providing the lowest^44^. In addition, calretinin expression evaluated by immunohistochemistry (IHC) in the three most frequent types of malignant mesothelioma separately present data that diverge from 18 to 100% positivity in sarcomatoid malignant mesothelioma, and 8 to 100% in biphasic malignant mesothelioma^45^. Therefore, differences may also exist in diagnostic efficiency of immunocytochemistry using calretinin when distinguishing certain subtype of MM from MC in serous effusions. In our study, only one study focused on the utility of calretinin for differential diagnosis between epithelioid MM and MC, thus further diagnostic tests in serous effusions should be performed to assess these differences among subtypes of MM.

Although we tried to avoid the biases in the process of meta-analysis, our study still had some limitations. First, only published studies were included in this meta-analysis, the exclusion of unpublished data, ongoing studies, conference abstracts and letters to editors may have led to publication bias. Second, the small sample-sized studies appeared to overestimate the true diagnostic accuracy of calretinin for the diagnosis of MM. Third, misclassification bias can occur since some adenocarcinoma was diagnosed in some patients based just on the clinical course, but not diagnosed by histological examination. This issue regarding accuracy of diagnosis can cause nonrandom misclassification, leading to biased results. Fourth, different cutoff values were used in the included studies, which made it difficult to determine the optimized cutoff value. Moreover, because of lack of required data reported in the original publications, we could not analyze the effect of factors such as laboratory infrastructure, expertise with tumor marker assay technology, patient spectrum and setting on the accuracy of the calretinin measurements. And, for the same reason, we could not explore whether the study design, such as blinded, cross-sectional, consecutive/random, and prospective design, affects the diagnostic accuracy, either.

It is also worth mentioning that up to now, limitations still exist in the diagnosis of MM by ICC in serous effusions. Cytologic examination of MM in routine practice is contributory to diagnosis only in the cases with adequate cytologic preparations^44^. Besides, in the era of precision medicine, obtaining histologic material is important for testing molecular alterations required for investigations in targeted therapies or immunotherapy of mesothelioma. In this case, biopsies and histologic assessment including immunohistochemistry still play a crucial role that cytologic examination cannot replace. Therefore, future works should focus on increasing the overall performance of cytologic diagnosis for MM to minimize the current weakness.

In conclusion, our meta-analysis is the first evidence-based study to date to have assessed the differential diagnostic utility of calretinin for MM in serous effusions. The results demonstrated that calretinin may be a useful adjunct to conventional diagnostic tools for accurately differentiating MM and MC, but should be interpreted in parallel with the gold standard of histological assessment and clinical findings when confirming diagnosis.

## Author Contributions

Conceived and designed the experiments: D.D.L., B.W. and F.Q.W. Performed the experiments: D.D.L. and H.Y.L. Analyzed the data: D.D.L. and B.W. Contributed reagents/materials/analysis tools: D.D.L., B.W. and H.Y.L. Wrote the manuscript: D.D.L., B.W. and F.Q.W.

## Figures and Tables

**Table 1:** Summary of the studies included in the meta-analysis

First author/year	Country	Method	Effusion type	Cut-off	Sample size	TP	FP	FN	TN	QUADAS score
Simisar A *et al.*, 1999	America	cell blocks	pleural	Cytoplasmic staining	55	15	9	11	20	10
Chhieng DC *et al.*, 2000	America	cell blocks	pleural and peritoneal	Cytoplasmic and nuclear staining	37	14	0	2	21	11
Kitazume H *et al.*, 2000	Japan	smears	pleural and peritoneal	≥1% cytoplasmic and/or nuclear stained cells	59	8	0	0	51	10
Davidson B *et al.*, 2001	Norway	cell blocks	pleural and peritoneal	Cytoplasmic and nuclear staining	110	11	3	1	95	11
Politi E *et al.*, 2005	Greece	smears	pleural, peritoneal and pericardial	>10% cytoplasmic and nuclear stained cells	84	4	16	0	64	10
Saad RS *et al.*, 2006	America	cell blocks	pleural	>5%moderate/strong cytoplasmic and nuclear stained cells	30	17	3	3	7	10
Bhalla R *et al.*, 2007	America	cell blocks	pleural and peritoneal	Cytoplasmic and nuclear staining	40	30	0	0	10	8
Hanley KZ *et al.*, 2007	America	cell blocks	pleural, peritoneal and pericardial	Cytoplasmic and nuclear staining	60	11	2	0	47	7
Lyons-Boudreaux V *et al.* 2008	America	cell blocks	pleural and peritoneal	Nuclear staining	53	5	1	0	47	6
Shield PW *et al.* 2008	Australia	cell blocks	pleural and peritoneal	>5% nuclear and cytoplasmic stained cells	101	33	2	1	65	8
Xu YM *et al.* 2008	China	cell blocks	pleural	Cytoplasmic and nuclear staining	52	6	1	0	45	9
Westfall DE *et al.* 2009	America	cell blocks	pleural	Cytoplasmic and nuclear staining	153	9	4	0	140	10
Ensani F *et al.* 2011	Iran	cell blocks	pleural and peritoneal	Cytoplasmic and nuclear staining	63	2	2	0	59	11
Su XY *et al.* 2011	China	cellblocks	pleural and peritoneal or pericardial	>5% nuclear and cytoplasmic stained cells	78	15	7	3	53	11
Hou G *et al.* 2012	China	smears	pleural	Cytoplasmic and nuclear staining	56	10	7	1	38	8
Hyun TS *et al.* 2012	America	cell blocks	pleural	Cytoplasmic staining	32	11	0	0	21	10
Luo L *et al.* 2013	China	cell blocks	peritoneal	≥10% cytoplasmic and/or nuclear stained cells	55	19	2	1	33	8
Chen JF *et al.* 2013	China	cell blocks	pleural	≥10% cytoplasmic and/or nuclear stained cells	1158	19	31	1	1107	11

TP, true positive; FP, false positive; FN, false negative; TN, true negative.

**Table 2:** Mata-regression of potential heterogeneity within the included studies

Covariates	Number of studies	Coefficient	SE	RDOR (95% CI)	*p* -value
Method					
Cell blocks	15	0.094	1.4303	1.10(0.05;23.16)	0.9486
Smeard	3				
Sample size					
≥100	4	1.813	1.0773	6.13(0.62–60.93)	0.1130
<100	14				
Geographical location					
America	9	0.118	0.54	1.12(0.36–3.56)	0.8306
Europe	3				
Asia	6				
QUADAS scores					
≥10	10	−1.962	0.9976	0.14(0.02–1.18)	0.0679
<10	8				

SE, Standard error.

**Table 3:** Summary of overall analysis and sensitivity analysis

Variables	Number of studies	Sensitivity (95% CI)	Specificity (95% CI)	DOR(95%CI)	PLR (95%CI)	NLR (95%CI)	AUC(95%CI)	Q-value
Overall analysis	18	0.91(0.87–0.94)	0.96(0.95–0.96)	163.03(54.62–486.63)	14.42(7.92–26.26)	0.1(0.05–0.2)	0.97	0.92
Cut-off of presenting nuclear staining	13	0.94(0.89–0.97)	0.94(0.91–0.95)	135.91(57.19–322.99)	13.41(7.58–23.72)	0.12(0.07–0.19)	0.97	0.92
Pleural effusion samples	7	0.84(0.76–0.91)	0.96(0.95–0.97)	91.84(12.42–679.04)	11.15(3.05–40.73)	0.12(0.03–0.47)	0.95	0.89

DOR, diagnostic OR; PLR, positive likelihood ratio; NLR, negative likelihood ratio; AUC, area under the receiver operating characteristic curve.
